# Regional disparities in cerebral perfusion and brain tissue microstructure damage in adult patients with Moyamoya syndrome

**DOI:** 10.1038/s41598-025-30486-4

**Published:** 2025-12-01

**Authors:** Lingyun Gao, Xiaoli Wang, Yuge Chen, Jingxuan Ning, Zhen Chong, Deguo Liu, Jiehuan Wang, Hao Yu, Yueqin Chen, Man Wang

**Affiliations:** 1 Department of Radiology, Affiliated Hospital of Jining Medical University, Jining, China; 2 Jining Medical University Clinical Medical College, Jining, China

**Keywords:** Moyamoya syndrome, Perfusion, Hemodynamics, Intravoxel incoherent motion, Microstructure, Neurology, Cerebrovascular disorders, Stroke, White matter disease

## Abstract

In Moyamoya syndrome (MMS), cerebral perfusion and tissue microstructure are impaired, but regional differences remain unclear. This study analyzed 22 adult MMS patients using CT perfusion (CTP) and intravoxel incoherent motion (IVIM). All hemispheres were classified into four ischemia grades based on symptoms and imaging. CTP parameters (CBF, CBV, MTT, TTP) and IVIM parameters (ADC, D, D*, f) were measured in the temporal lobe and basal ganglia. The relative values of CTP parameters and absolute values of IVIM parameters were compared across hemispheres with different ischemia grades. Additionally, correlation analyses were conducted between CTP and IVIM parameters. Results showed that in the temporal lobe, rMTT and rTTP were significantly increased (*p* = 0.018 and 0.002, respectively) with higher ischemia grades, while basal ganglia changes were similar but milder, with only rTTP delay being significant (*p* = 0.011). IVIM analysis revealed significantly elevated ADC values in association with higher ischemia grades (overall *p* = 0.011). Additionally, significant variations were observed in both D* (overall *p* = 0.041) and the perfusion fraction f (overall *p* = 0.043). In the basal ganglia, IVIM parameters showed no significant differences across ischemia grades. Weak or insignificant correlations were found between IVIM parameters and CTP parameters. The study suggested that perfusion and microstructural damage in the basal ganglia were less severe than in the temporal lobe, and that IVIM offers insights into microvascular status that are complementary to those from hemodynamic perfusion imaging.

**Supplementary Information:**

The online version contains supplementary material available at 10.1038/s41598-025-30486-4.

## Introduction

Moyamoya syndrome (MMS) is a cerebrovascular disease characterized by progressive stenosis and/or occlusion of the terminal portion of the internal carotid artery and/or the origin of the anterior and/or middle cerebral artery, along with the formation of collateral vessels at the base of the brain^1,2^. Due to the narrowing or occlusion of the major intracranial arteries, patients with MMS suffer from impaired cerebral blood flow dynamics, which can lead to varying degrees of ischemic symptoms^3,4^. Moreover, the long-term chronic ischemia can cause varying degrees of damage to the brain tissue microstructure, which may be related to their cognitive function status in patients with MMS^5–8^. Therefore, the evaluation of brain tissue perfusion and microstructural changes in patients with MMS is a crucial step, which aids in determining the timing of surgery and the prediction of prognosis^9–11^.

Due to the complexity of cerebrovascular stenosis and collateral circulation, the hemodynamic and microstructural changes in different brain regions of the anterior circulation in patients with MMS are inconsistent^12–14^. However, there is still a lack of comparative studies on the differences in hemodynamics and microstructure of brain tissue between different brain regions in MMS, especially between the cerebral cortex and central brain areas, such as the temporal lobe and the basal ganglia^13,15,16^. Understanding the differences in blood perfusion between various brain regions and microstructural damage to brain tissue will aid in the comprehensive assessment of patients with MMS, providing a reference for the timing of surgery and the choice of surgical procedures.

CT perfusion imaging is one of the most commonly used non-invasive methods to evaluate cerebral hemodynamics^17–19^, enabling quantitative analysis of the blood flow perfusion status of damaged brain tissue through parameters such as cerebral blood flow (CBF), cerebral blood volume (CBV), mean transit time (MTT), and time to peak (TTP). Intravoxel incoherent motion (IVIM) imaging is a MRI technique that proposes the simultaneous measurement of microvascular and parenchymal microstructural tissue properties without gadolinium administration. IVIM based on multiple b-values enables the separation of the intravoxel signal into diferent quantitative perfusion-related contributions and diffusion parameters due to Brownian motion, such as the perfusion fraction (f), diffusion (D), pseudo-diffusion (D*), and apparent diffusion coefficient (ADC)^20–23^.

Therefore, this study aims to include patients with MMS who have undergone both CT perfusion and IVIM examinations to explore the disparity in hemodynamics and microstructural changes between the basal ganglia and the temporal lobe.

## Methods

### Study design and population

Between July 2021 and May 2024, consecutive MMS patients diagnosed by MRI/MRA according to the criteria of the Research Committee on Spontaneous Occlusion of the Circle of Willis of the Ministry of Health and Welfare, Japan (2022) at the Affiliated Hospital of Jining Medical University were retrospectively reviewed. Considering the retrospective design and anonymized data utilized, the local ethics committee of Affiliated Hospital of Jining Medical University approved a waiver of informed consent requirements. We have used the term MMS in all cases, whether or not there was an associated diagnosis, to describe the characteristic vasculopathy^24^. The inclusion criteria were as follows: (1) ≥ 18 years old; (2) underwent both intracranial CTP and IVIM examinations. The exclusion criteria were as follows: (1) hemorrhagic MMS; (2) the interval between CTP and IVIM examinations was more than 1 month; (3) patients who have underwent revascularization; (4) image quality of CTP and IVIM was severely impaired. The study protocol was approved by the hospital ethics committee and written informed consent was waived.

### Imaging protocols

CTP was performed with a Siemens Somatom Definition Flash CT scanner (Siemens Healthineers, Erlangen, Germany) as previously described^24^. Briefly, following the injection of 50 mL of contrast medium (Ultravist 300; Bayer Schering, Berlin, Germany) at a rate of 5 mL/s, dynamic acquisition was initiated after a 7-second delay. The acquisition parameters were as follows: 80 kV, 200 mAs, 0.75-mm slice thickness, 128 × 0.6-mm collimation, 0.28-s rotation time, and a total imaging time of 53 s. A z-axis coverage of approximately 10.0 cm was achieved using the adaptive spiral scanning technique (‘shuttle mode’). For perfusion analysis, a set of axial images with a slice thickness of 10.0 mm was reconstructed without overlap. All MR examinations were performed with a 3-T whole-body MRI scanner (Discovery MR750, GE Medical Systems, Chicago, IL, USA) with a 32-channel receiver head coil. IVIM imaging was based on a diffusion-weighted spin-echo echo planar imaging (EPI) sequence, with 11 b-values (0, 10, 20, 50, 80, 100, 200, 500, 800, 1000, and 2000 s/mm^2^) in the scan sequence. Other parameters were as follows: 5 mm section thickness; 240 × 240 mm^2^ field of view; 160 × 160 matrix size; 4000 ms repetition time (TR); minimum echo time (TE); 3 min 20 s acquisition time.

### Imaging post-processing

All reconstructed axial CTP images were transferred to a workstation (Syngo MMWP, VE 40 C, Siemens Healthcare). Perfusion analysis was performed for all datasets with the vendor given “Neuro-VPCT” software, using the semiautomatic deconvolution algorithm “Auto Stroke MTT”. The first artery to reach peak enhancement on the time-attenuation curve was selected as the arterial input function. The venous input region of interest (ROI) was placed in the superior sagittal sinus. Perfusion parameter maps for CBF, CBV, MTT and TTP were generated.

**Fig. 1:**
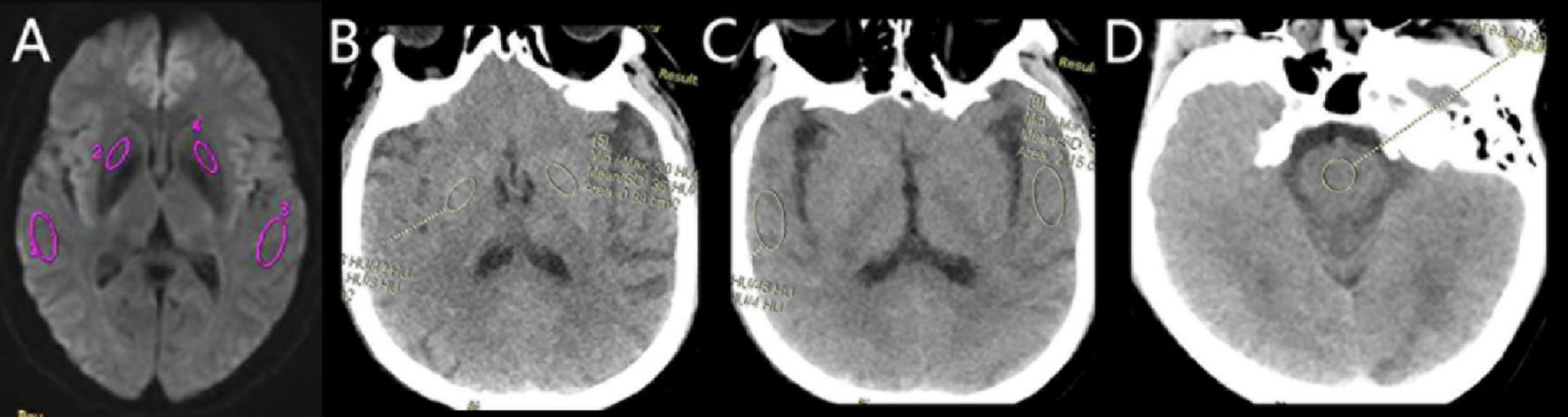
Schematic illustration of region of interest (ROI) placement. Standardized ROIs were manually drawn on the (**A**) temporal lobe, (**B**) basal ganglia (lenticular nucleus), and (**C**) pons. Panels A and B show ROIs on CT perfusion source images, while panel C shows a corresponding ROI on an IVIM parametric map.

The IVIM and DKI images were transferred to the GE AW4.6 workstation (version 9.4.05, GE Healthcare), and all images were processed and analyzed using a relevant software in the functional kit. The IVIM parameters were calculated using the following Eqs^25,26^.: S_b_/S_0_ = (1 − *f*) exp (− *b*D) + *f* exp[− *b*(D*+D)]. This biexponential model was used to calculate ADC, D, D* and f values. The region of interest (ROI) was delineated on the grayscale map with a b-value of 1000 s/mm^2^. The color-coded parametrical maps of the IVIM were then merged with the grayscale map using the 3D-SynchroView (GE Healthcare). Standardized ROIs were manually drawn on the bilateral temporal lobes, basal ganglia (lenticular nucleus), and on CTP images, the pons (Fig. 1), which served as a relatively stable reference region given its supply from the vertebrobasilar system and typical sparing from MMS^27,28^. The placement followed a detailed protocol with the following specifics: oval ROIs of ~ 2 cm² were placed in the temporal lobe parenchyma, oval ROIs of ~ 1 cm² were centered on the lenticular nucleus, and circular ROIs of ~ 1 cm² were positioned in the pons, with care taken to avoid large vessels, CSF spaces, and visible infarcts. All measures were operated by two experienced (more than 10 years experience) neuroradiologists. The relative CTP values in our study were defined as the ratios between the absolute CTP values of the temporal lobe or basal ganglia and those of the pons area. The average values were calculated and recorded for statistical analysis. Inter-observer consistency was quantitatively assessed using the intraclass correlation coefficient (ICC).

### Data collection

Demographic data and routine imaging characteristics were recorded. Based on the patients’ symptoms and imaging findings, all included patients were categorized into three ischemia grades (asymptomatic type, atypical type, and typical ischemia type). Additionally, at the level of the cerebral hemispheres, all cerebral hemispheres of included patients were classified into four ischemia grades (normal, asymptomatic type, atypical type, and typical ischemia type). The criteria for determining the type of ischemia were as follows^29^: Typical ischemia type was defined as focal neurological deficits with magnetic resonance diffusion-weighted imaging (DWI) showing recent infarct lesions or classic symptoms of transient ischemic attack (TIA) combined with involvement of major vessels in the culprit hemisphere. Atypical type was defined as dizziness, headache, facial or limb discomfort, but not meeting the diagnostic criteria for TIA or cerebral infarction. The determination of asymptomatic type was divided into two scenarios. The first was at the patient level, where the patient was diagnosed with MMS (no matter unilateral or bilateral) during a routine examination, presenting with no clinical symptoms. The second was at the level of the cerebral hemisphere, characterized by the involvement of major vessels on that side of the brain, but without clinical symptoms or infarction lesions related to that hemisphere (Table S1). In this study, the normal type was defined as the cerebral hemisphere with normal major vessels in patients with unilateral MMS.

### Statistical analysis

All discrete or categorical variables were presented as numbers and relative.

frequencies (percentages) and continuous variables as mean ± standard deviation or median with interquartile range normally distributed data. The inter-observer agreement for the quantitative measurements of both rCTP and IVIM parameters was evaluated using the ICC, interpreted as follows: <0.50, poor; 0.50–0.75, moderate; 0.75–0.90, good; and > 0.90, excellent reliability. Comparisons of continuous variables were analysed by ANOVA test or Kruskal-Wallis test among different ischemia grades according to the data distribution at the hemisphere level. Comparisons of categorical variables between groups were performed by Chi-square test or Fisher’s exact test, as appropriate. The correlation analysis between the parameters of IVIM and CT perfusion were conducted. Correlations for variables that were not normally distributed were assessed using Spearman rank order correlation plotted in a heatmap. Correlation coefficients of < 0.2 were regarded as very weak, 0.2 to < 0.40 as weak, 0.40 to < 0.60 as moderate, 0.6 to < 0.80 as strong, and 0.8 to 1 as very strong. A statistically significant difference was defined as a 2-sided *p* value < 0.05. Statistical analysis was performed using R, version 4.2.3 (R Foundation for Statistical Computing, Vienna, Austria).

## Results

### Characteristics of patients and hemispheres

**Fig. 2:**
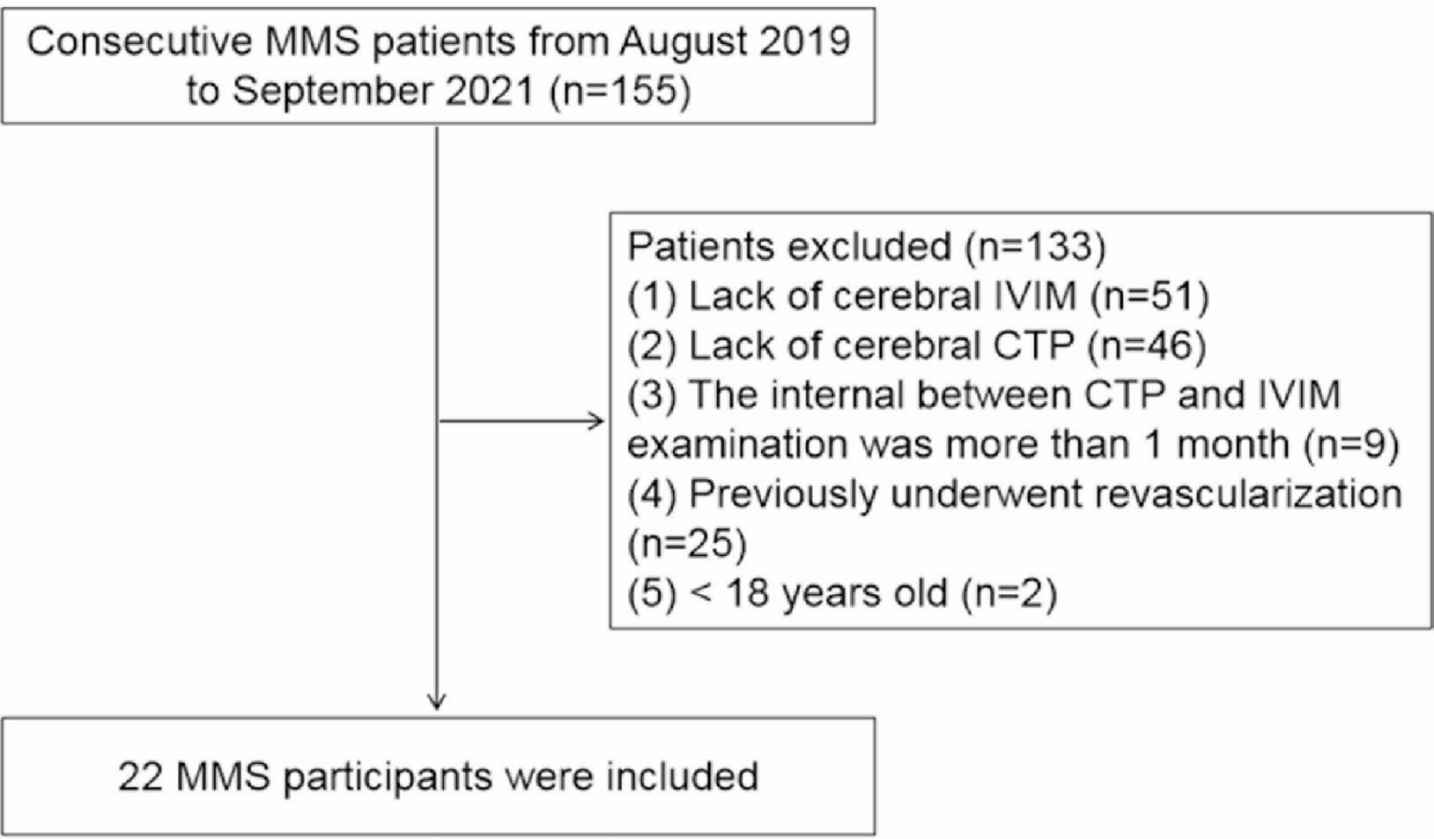
Flowchart of the enrolled MMS patients.

This study included 22 MMS patients where CTP and IVIM images were of suitable image quality for analysis (Fig. 2). The mean age of these patients was 51 ± 7 years, 50% were male. Among 22 MMS patients, 11 were diagnosed with unilateral MMS and 11 were bilateral MMS patients. The median time interval between the CTP and IVIM examinations was 2.5 days (interquartile range: 1.0–9.8 days). The ischemia grades of the patients and their corresponding cerebral hemispheres are shown in Table 1. The procedures of this study followed were in accordance with the ethical standards of the responsible committee on human experimentation (institutional or regional) and with the Helsinki Declaration of 1975, as revised in 2000.

**Table 1:** Clinical characteristics of the MMS patients in the study.

Characteristic	Overall (*n* = 22)
Age, y	50.91 ± 7.04
Sex	
Female	12 (54.55%)
Male	10 (45.45%)
Affected hemispheres (patient level)	
Unilateral MMS	11 (50.00%)
Bilateral MMS	11 (50.00%)
Ischemia grade (patient level)	
1	4 (18.18%)
2	9 (40.91%)
3	9 (40.91%)
Ischemia grade (hemisphere level)	
0	7(15.91%)
1	15(34.09%)
2	13(29.55%)
3	9(20.45%)

Values are mean (standard deviation) or n (%). MMS: Moyamoya syndrome. 0: normal type; 1: asymptomatic type; 2: atypical type; 3: typical ischemia type.

### Differences in CTP parameters among different ischemia grades (hemisphere level)

**Fig. 3:**
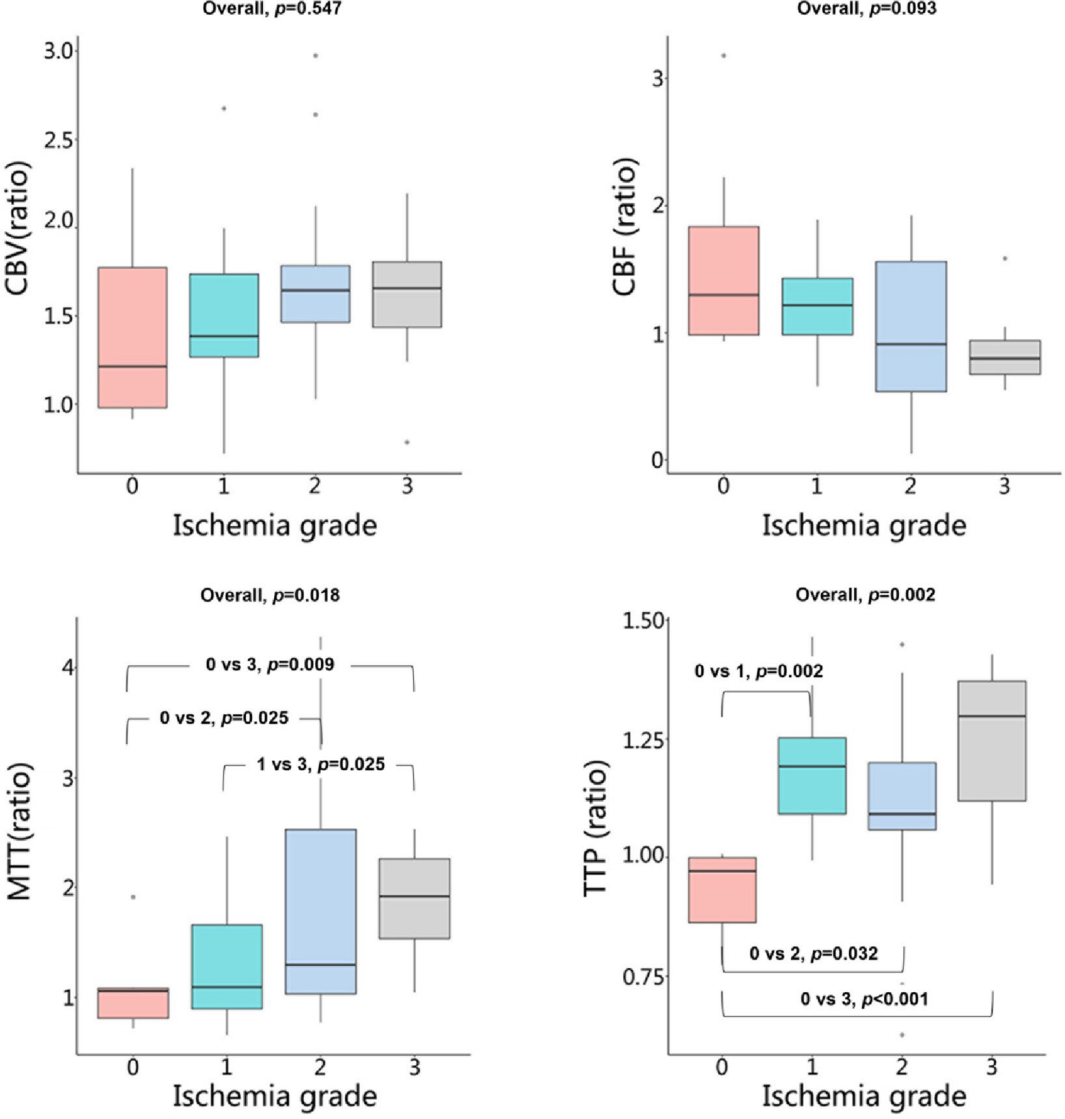
Box plots of CTP parameters (rCBV, rCBF, rMTT, and rTTP) in the temporal lobe across different cerebral ischemia grades. Overall *p *values from group comparisons are shown for each parameter. CBV, cerebral blood volume; CBF, cerebral blood flow; MTT, mean transit time; TTP, time to peak

**Fig. 4:**
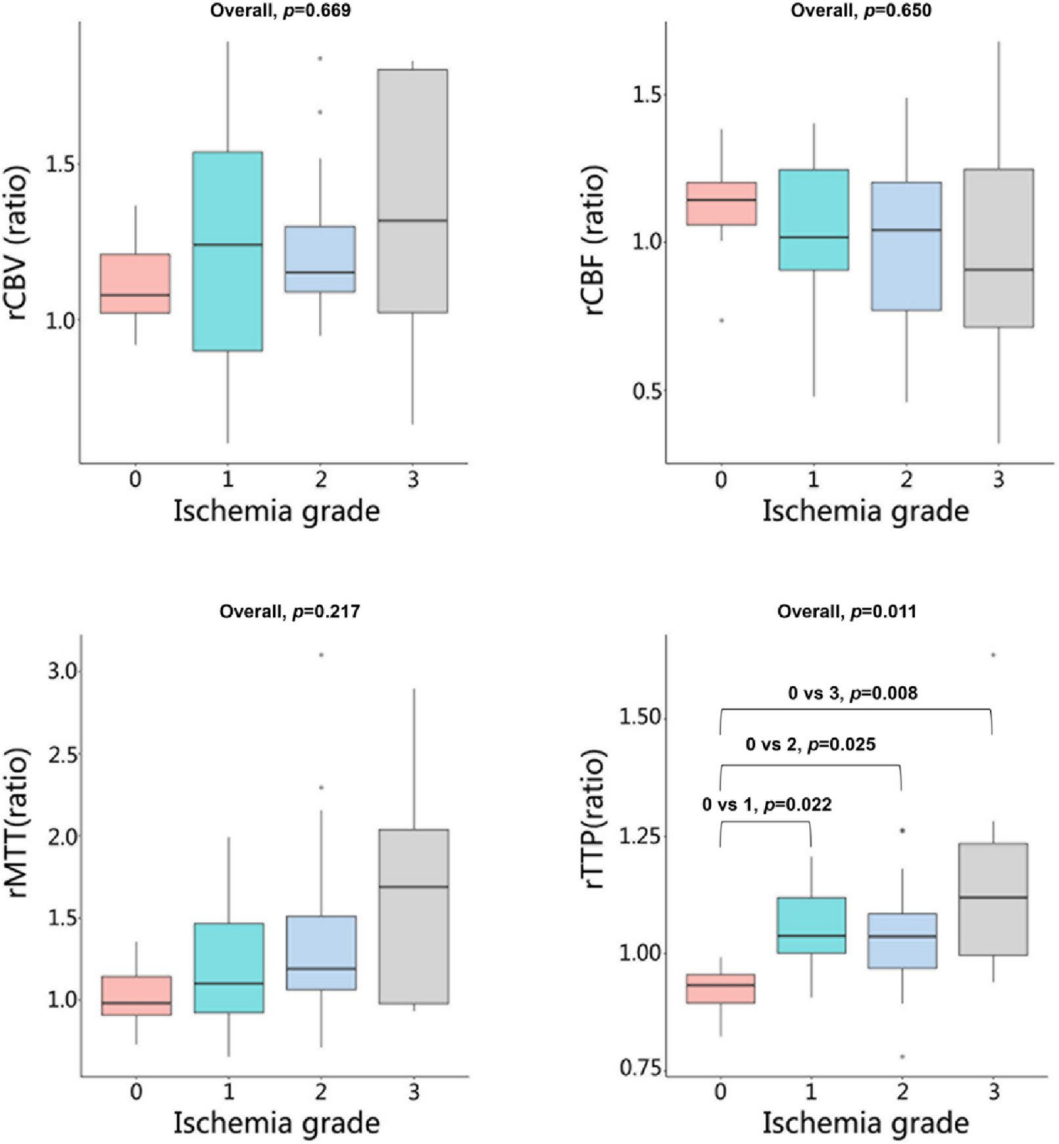
Box plots of CTP parameters (rCBV, rCBF, rMTT, and rTTP) in the basal ganglia across different cerebral ischemia grades. Overall*p *values from group comparisons are shown for each parameter. CBV, cerebral blood volume; CBF, cerebral blood flow; MTT, mean transit time; TTP, time to peak.

The inter-observer reliability for all quantitative rCTP and IVIM parameters was excellent, with ICCs ranging from 0.81 to 0.91 (Table S2 and Table S3). Regarding cerebral perfusion, significant differences between the temporal lobe and basal ganglia were observed as ischemia severity increased (Table 2, Table S4, Figs. 3 and 4). In the temporal lobe, rMTT and rTTP were significantly prolonged (Overall *p* = 0.018 and *p* = 0.002, respectively). Post-hoc tests for rTTP showed significant delays in grades 1, 2, and 3 compared to grade 0 (all *p* values < 0.05). In contrast, trends of increasing rCBV and decreasing rCBF were not statistically significant (*p* = 0.547 and *p* = 0.093). In the basal ganglia, changes in CTP parameters were less pronounced. Only rTTP demonstrated a significant overall increase (Overall *p* = 0.011), with grades 1, 2, and 3 all being significantly delayed compared to grade 0 (all *p* values < 0.05). No significant differences were found in rCBV, rCBF, or rMTT (all *p* values > 0.65). In summary, perfusion timing parameters (especially rTTP) were more severely affected in the temporal lobe, while the basal ganglia exhibited a more limited and delayed hemodynamic disturbance.

**Table 2:** Differences in CTP parameters among different ischemia grades stratified by the Temporal lobe and basal ganglia (cerebral hemisphere level).

Parameter	Ischemia grade				H value	*P* value
	0	1	2	3		
Number	7	15	13	9		
Temporal lobe						
rCBV	1.21 (0.98–1.77)	1.38 (1.27–1.74)	1.64 (1.46–1.78)	1.66 (1.43–1.81)	1.637	0.547
rCBF	1.30 (0.98–1.84)	1.22 (0.98–1.43)	0.91 (0.54–1.56)	0.80 (0.67–0.94)	6.414	0.093
rMTT	1.06 (0.81–1.08)	1.09 (0.89–1.66)	1.29 (1.03–2.53)	1.92 (1.53–2.26)	10.060	0.018
rTTP	0.97 (0.86–1.00.86.00)	1.19 (1.09–1.25)	1.09 (1.06–1.20)	1.30 (1.12–1.37)	14.507	0.002
Basal ganglia						
rCBV	1.08 (1.02–1.21)	1.24 (0.90–1.54)	1.15 (1.09–1.30)	1.32 (1.02–1.80)	1.281	0.669
rCBF	1.14 (1.06–1.20)	1.02 (0.91–1.24)	1.04 (0.77–1.20)	0.91 (0.71–1.25)	1.725	0.650
rMTT	0.98 (0.91–1.14)	1.10 (0.92–1.47)	1.19 (1.06–1.51)	1.69 (0.98–2.04)	4.443	0.217
rTTP	0.93 (0.89–0.96)	1.04 (1.00–1.12.00.12)	1.04 (0.97–1.09)	1.12 (1.00–1.24.00.24)	11.201	0.011*

Values distributed non-normally are presented as median (upper and lower quartiles). 0: normal type; 1: asymptomatic type; 2: atypical type; 3: typical ischemia type; rCBV: relative cerebral blood volume; rCBF: relative cerebral blood flow; rMTT: relative mean transit time; rTTP: relative time to peak; N: number; CTP: CT perfusion; * : *p* < 0.05.

### Differences in IVIM parameters among different ischemia grades (hemisphere level)

**Fig. 5:**
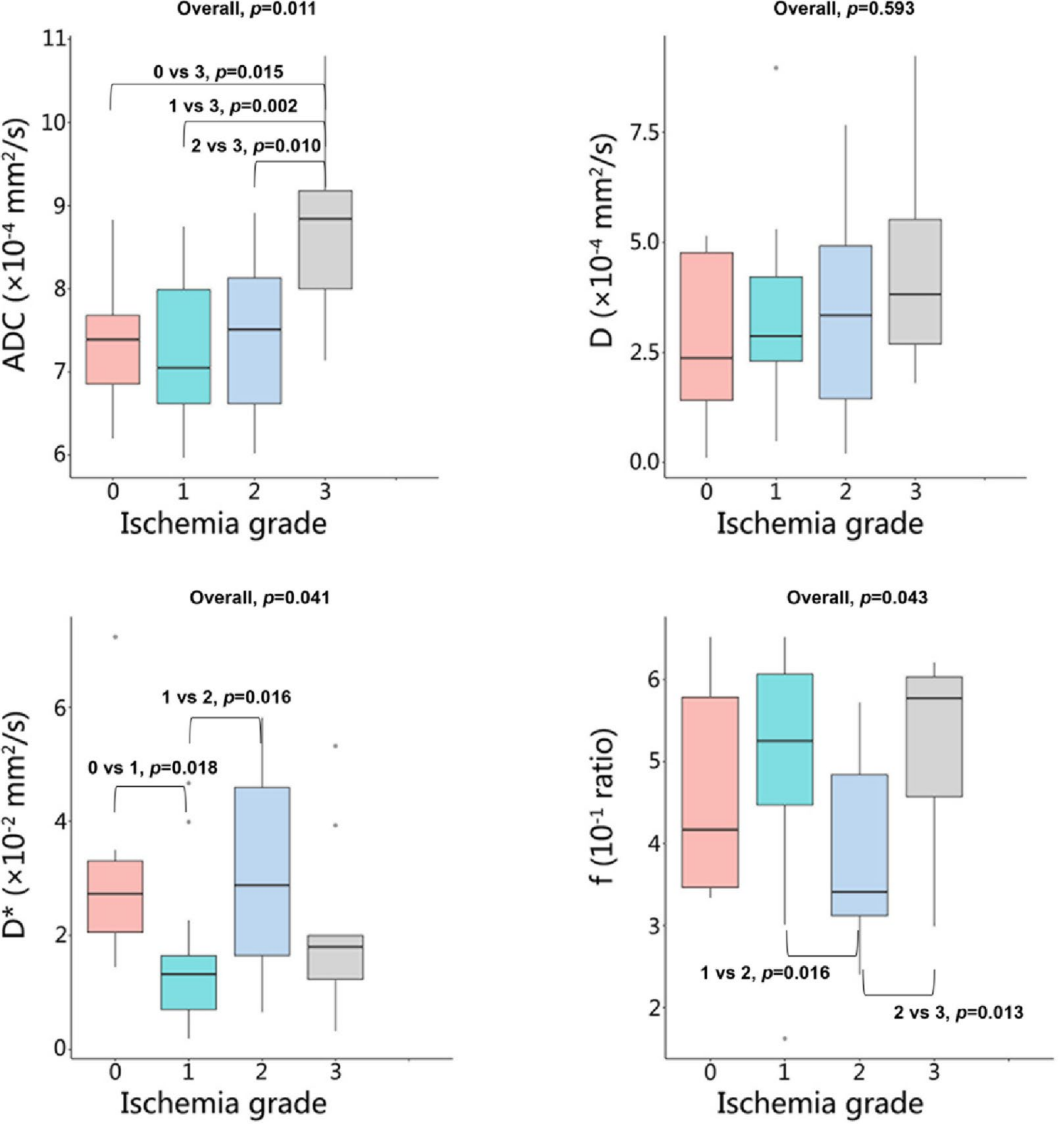
Box plots of IVIM parameters (ADC, D, D*, and f) in the temporal lobe across different cerebral ischemia grades. Overall*p *values from group comparisons are shown for each parameter. ADC, apparent diffusion coefficient; D, diffusion; D*, pseudo-diffusion; f, perfusion fraction.

**Fig. 6:**
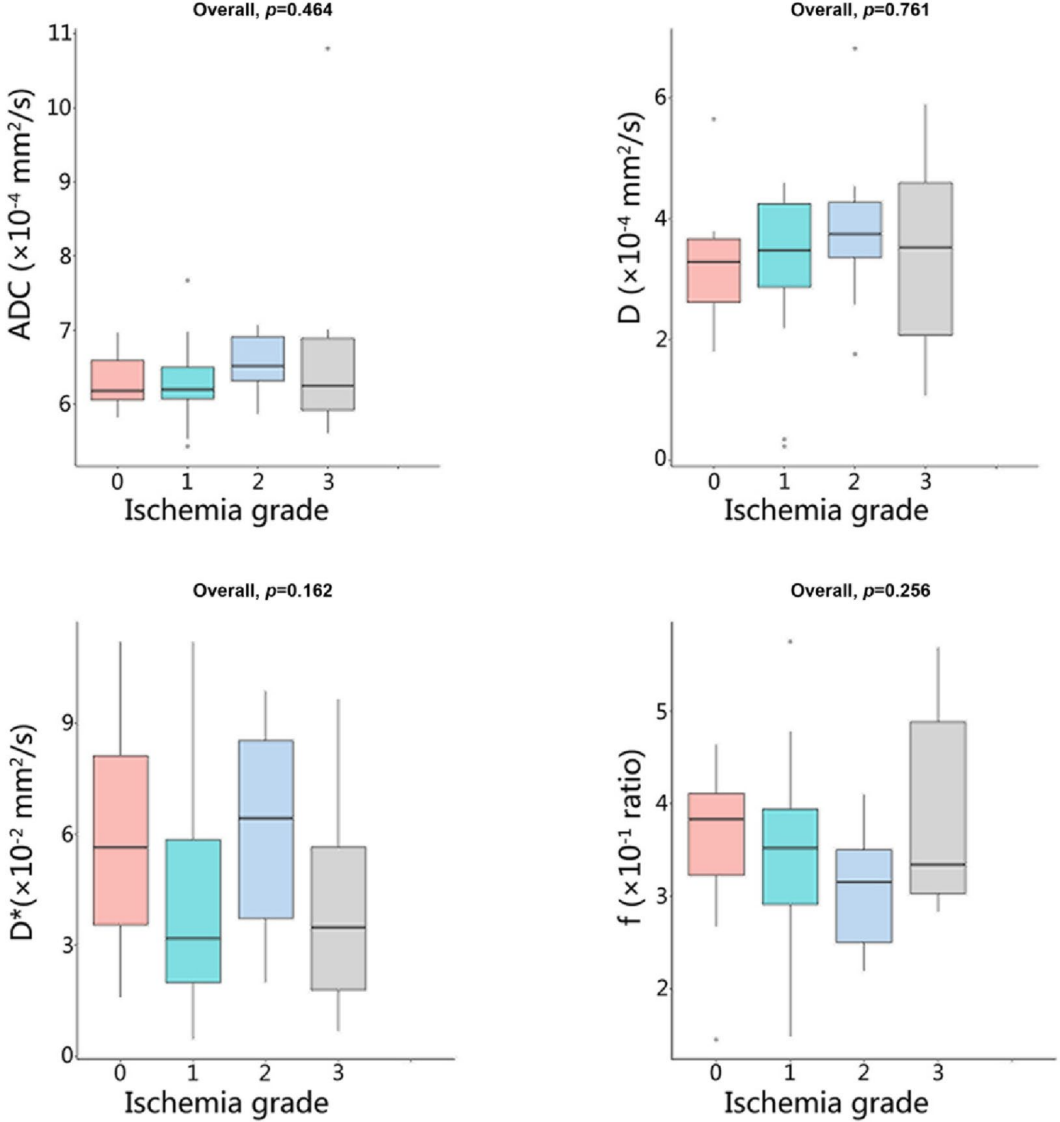
Box plots of IVIM parameters (ADC, D, D*, and f) in the basal ganglia across different cerebral ischemia grades. Overall*p *values from group comparisons are shown for each parameter. ADC, apparent diffusion coefficient; D, diffusion; D*, pseudo-diffusion; f, perfusion fraction.

Analysis of IVIM parameters revealed distinct patterns in the temporal lobe (Table 3, Table S4, Figs. 5 and 6). The ADC values showed a significant increasing trend with higher ischemia grades (Overall *p* = 0.011), with post-hoc tests confirming significantly higher values in grade 3 compared to grades 0, 1, and 2 (all *p* values < 0.05). Significant overall differences were also observed for D* and f (*p* = 0.041 and *p* = 0.043, respectively). Post-hoc analysis for D* indicated significant differences between several groups, most notably lower values in grade 1 compared to grade 0 and grade 2. Similarly, f differed significantly, primarily driven by higher values in grade 2 compared to grades 1 and 3. In contrast to the temporal lobe, none of the IVIM parameters demonstrated significant differences across ischemia grades within the basal ganglia region (all Overall *p* values > 0.05).

**Table 3:** Differences in IVIM parameters among different ischemia grades stratified by the Temporal lobe and basal ganglia (cerebral hemisphere level).

Parameter	Ischemia grade				H value	*P* value
	0	1	2	3		
Number	7	15	13	9		
Temporal lobe						
ADC (×10^− 4^ mm^2^/s)	7.39 (6.86–7.68)	7.05 (6.62–7.99)	7.51 (6.62–8.13)	8.84 (8.00–9.18.00.18)	11.229	0.011*
D (×10^− 4^ mm^2^/s)	2.37 (1.41–4.76)	2.87 (2.31–4.21)	3.34 (1.45–4.92)	3.82 (2.69–5.52)	1.900	0.593
D* (×10^− 2^ mm^2^/s)	2.73 (2.05–3.30)	1.32 (0.70–1.64)	2.88 (1.65–4.59)	1.80 (1.23–2.00.23.00)	8.272	0.041*
f (×10^− 1^)	4.17 (3.47–5.79)	5.25 (4.47–6.06)	3.41 (3.12–4.84)	5.77 (4.57–6.03)	8.137	0.043*
Basal ganglia						
ADC (×10^− 4^ mm^2^/s)	6.18 (6.06–6.59)	6.20 (6.07–6.50)	6.52 (6.32–6.91)	6.25 (5.92–6.89)	2.563	0.464
D (×10^− 4^ mm^2^/s)	3.29 (2.61–3.66)	3.48 (2.87–4.25)	3.75 (3.36–4.27)	3.53 (2.07–4.60)	1.166	0.761
D* (×10^− 2^ mm^2^/s)	5.64 (3.54–8.12)	3.18 (1.99–5.84)	6.43 (3.73–8.52)	3.48 (1.79–5.65)	5.141	0.162
f (×10^− 1^)	3.83 (3.22–4.11)	3.52 (2.91–3.94)	3.15 (2.50–3.50)	3.34 (3.03–4.88)	4.054	0.256

Values distributed non-normally are presented as median (upper and lower quartiles). 0: normal type; 1: asymptomatic type; 2: atypical type; 3: typical ischemia type; IVIM: intravoxel incoherent motion; f: perfusion fraction; D: diffusion; D*: pseudo-difusion; ADC: apparent difusion coefcient. N: number; * : *p* < 0.05.

### Correlation analysis between the parameters of IVIM and CTP

**Fig. 7:**
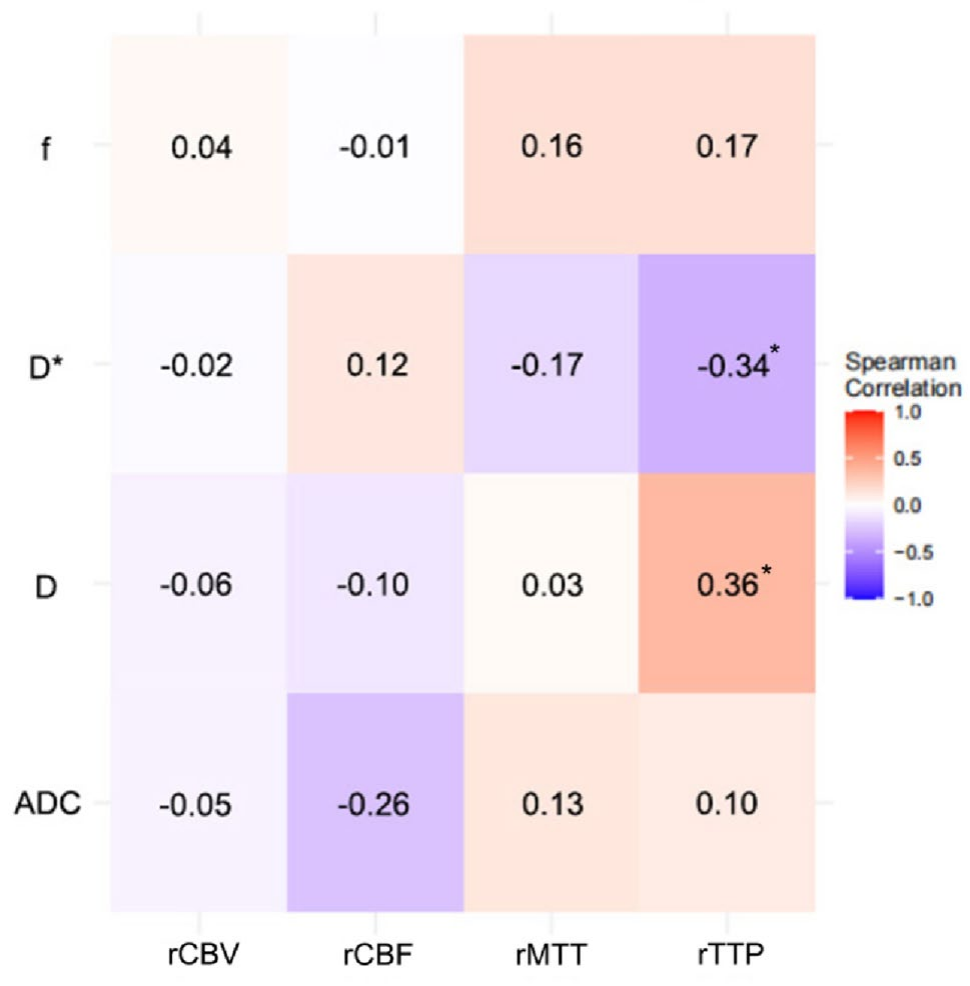
Spearman correlation heatmap between perfusion parameters (rCBV, rCBF, rMTT, rTTP) and IVIM parameters (ADC, D, D*, f) in the temporal lobe. CBV, cerebral blood volume; CBF, cerebral blood flow; MTT, mean transit time; TTP, time to peak; ADC, apparent diffusion coefficient; D, diffusion; D*, pseudo-diffusion; f, perfusion fraction; * indicates *p* <0.05.

**Fig. 8:**
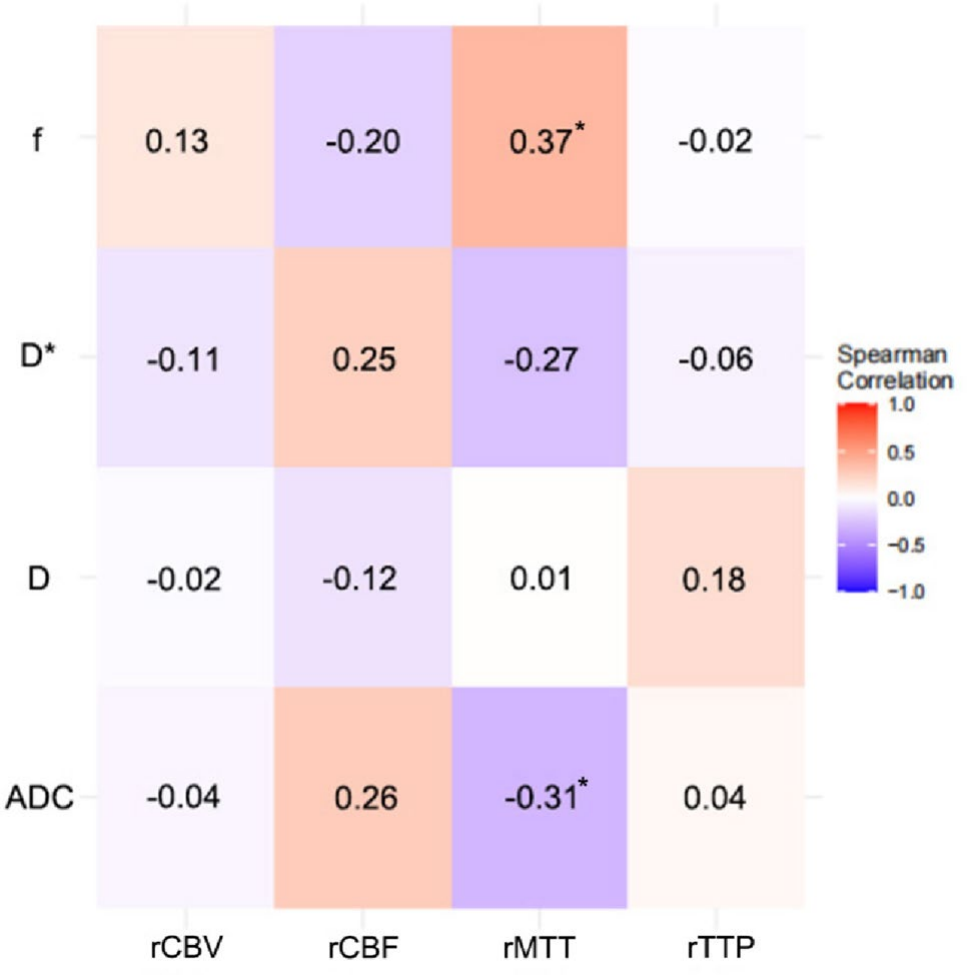
Spearman correlation heatmap between perfusion parameters (rCBV, rCBF, rMTT, rTTP) and IVIM parameters (ADC, D, D*, f) in the basal ganglia. CBV, cerebral blood volume; CBF, cerebral blood flow; MTT, mean transit time; TTP, time to peak; ADC, apparent diffusion coefficient; D, diffusion; D*, pseudo-diffusion; f, perfusion fraction; * indicates *p* <0.05.

To validate the correlation between CTP and IVIM parameters, we conducted a series of correlation analyses. The results revealed that in the temporal lobe region, only the D and D* values showed a weak correlation with rTTP (*r* = 0.36, *p* = 0.016; *r*=−0.34, *p* = 0.025; respectively). In the basal ganglia region, only the ADC and f values exhibited a weak correlation with rMTT (*r*=−0.31, *p* = 0.037; *r* = 0.37, *p* = 0.014; respectively). No significant statistical correlation was found between other CTP parameters and IVIM parameters (Figs. 7 and 8).

## Discussion

The present study had 3 major findings. Firstly, in comparison with basal ganglia, temporal lobe was more likely to suffer hemodynamic and microstructural damage. And, the higher the ischemia grade, the more severe the perfusion and microstructural damage in the temporal lobe. Secondly, ADC, D*, and f were sensitive parameters of IVIM to detect the microstructural damage. Additionally, the correlations between the parameters of IVIM and CTP were weak, which indicated that IVIM-derived parameters offer complementary and unique information regarding tissue microvascular status beyond what is captured by traditional hemodynamic perfusion imaging.

In patients with MMS, the hemodynamic damage in the anterior circulation areas of the brain is often more severe, especially in the temporal lobe^28,30,31^. However, the blood perfusion in the basal ganglia region differs, as this area is primarily supplied by penetrating vessels such as the lenticulostriate arteries^32,33^. In MMS, these penetrating vessels significantly increase and dilate, which may to some extent ensure the blood perfusion of the basal ganglia region^14,34^. This study showed that as the grade of ischemia increased, the rCBV of the temporal lobe brain tissue gradually increased and the rCBF gradually decreased, meanwhile, the rTTP and rMTT were significantly delayed. These results indicated that the microcirculation in the temporal lobe brain area was significantly dilated and congested, with relatively severe perfusion injury. However, in the basal ganglia region, with the increase in the grade of ischemia, although the trend of changes in all CTP parameters was the same as that in the temporal lobe, only the rTTP parameter showed a significant statistical difference. This proved the theory that the degree of cerebral perfusion injury in the basal ganglia region was lower than that in the temporal lobe.

Previous studies showed that MMS was often accompanied by microstructural damage to brain tissue, which can be indicated by an increased ADC^11,35–37^. This study demonstrated the same results, showing that ADC values tended to increase as the grade of ischemia increased. However, traditional diffusion-weighted ADC has been demonstrated to be influenced not only by molecular diffusion but also by blood perfusion of microcirculation. Therefore, the reliability of traditional ADC may be limited in characterizing microstructural damage to brain tissue. It has also been shown that microcirculation or perfusion effects can be distinguished from true tissue diffusion by using multiple b value sampling and a biexponential curve fit analysis with the IVIM model^21,38^. This study showed that as the grade of ischemia increased, the D values of the temporal lobe gradually increased, but there were no significant statistical differences. It was noteworthy that as the grade of ischemia increased, the D* values in the temporal lobe exhibited a wave-like decreasing trend; in contrast, the f values showed a wave-like increasing trend, and both differences were statistically significant. The phenomenon may be explained by the compensatory hemodynamic mechanisms. Previous studies suggest that D* and f could indirectly reflect microcirculation, which are related to the 1/MTT (or 1/Tmax) and CBV parameters of CTP, respectively^39,40^. During the asymptomatic or atypical symptom phase, brain perfusion was in a compensatory period, and D* might have remained stable or increased. In the typical ischemic phase, D* values significantly decreased, which was exactly the opposite of the changes in rTTP (Fig. 5), consistent with previous studies^39^. However, the trend of changes in f values was not the same as that of D*, possibly influenced by the complexity of microcirculation, similar to rCBV. This study found that as the grade of ischemia increased, D* in the basal ganglia also showed a wave-like decreasing trend, but the changes in ADC, D, and f were relatively weak (all *p* values > 0.05). Therefore, the results of this study indicated that, compared to the temporal lobe, MMS exhibited a sign of “basal ganglia preservation”.

IVIM emerged as a different approach for the obtaining of perfusion information and studies have aimed to correlate IVIM outputs with classic perfusion-related measures^41^. In a study of patients with acute cerebral infarction, Guangming Zhu et al.^39^found that the f parameter and D* parameter showed similar infarct volumes to the CBV and MTT parameters in MR perfusion, respectively. However, the study only conducted a Bland-Altman analysis on the infarct volumes shown by the two methods, without performing a correlation analysis on the IVIM and MR perfusion parameter values themselves. Y. Yao et al^42^. conducted a comparative study on IVIM and CBF based on arterial spin labeling perfusion (ASL) in patients with cerebral infarction, and the results revealed that only the f parameter was mildly correlated with the CBF parameter. In a study of patients with Moyamoya disease, Koji Yamashita et al.^15^ found that the f parameter in the basal ganglia region showed a mild negative correlation with CBF based on SPECT examination. However, the study also found that the correlation between the f parameter and CBF parameters was not statistically significant in non-Moyamoya disease patients. Meanwhile, although the f parameter is considered to be related to CBV, yet there are few studies that have found a correlation between these two parameters. Therefore, it can be seen from previous studies that the correlation between IVIM parameters and classical perfusion parameters is generally low or insignificant. The results of this study showed that the correlation between each parameter of IVIM with various relative CT perfusion parameters was weak. These findings can be interpreted from two complementary perspectives. First, a fundamental biophysical divergence exists: as originally proposed by Denis Le Bihan, the IVIM “perfusion” signal reflects the radiologist’s perspective of blood flow within the vascular compartment, distinct from the physiological perspective of blood flow supplying the tissue^43^. In MMS, this means CTP captures the compromised net inflow from large artery stenosis, whereas IVIM is sensitive to the architectural and flow characteristics of the adapting microvascular bed. Thus, IVIM provides a unique window into microvascular architecture rather than a direct measure of tissue perfusion. Second, methodological considerations, such as spatial and temporal misalignment between non-simultaneous MRI and CT acquisitions, may also attenuate correlations. Therefore, despite its longer acquisition time, IVIM offers unique clinical value as a non-contrast and radiation-free technique, making it suitable for vulnerable populations or serial monitoring. In contrast, CTP remains advantageous for rapid assessment in emergency settings. Rather than serving as a substitute, IVIM thus provides complementary insights into microvascular status, offering a unique perspective on the underlying tissue environment.

Notably, collateral circulation is vital for maintaining cerebral perfusion in MMS, as its patterns are closely linked to clinical outcomes. For example, anterior choroidal artery dilation increases hemorrhage risk^44^, whereas posterior circulation involvement is associated with worsened functional outcomes^45^. Thus, variations in collateral development may be a key mechanism underlying the regional differences in CTP and IVIM parameters observed in our study. Future work should include detailed collateral assessments to better elucidate its role in MMS hemodynamics and microstructural damage.

This study has several limitations. First, the sample size was relatively small. To address this, analyses were conducted at the hemispheric level and a post-hoc power analysis was performed, which confirmed the ability to detect alterations with large effect sizes (Table S5 and Table S6). However, larger studies are needed for validation. Second, the retrospective design carries a risk of selection bias, despite the implementation of strict exclusion criteria (e.g., prolonged interval between scans or prior revascularization). Third, relative CTP values were derived using the pons as a reference region. Although the pons is frequently used as a reference in MMS studies, this approach carries an inherent risk. Fourth, the ischemia grading was based on clinical symptoms, which involves an element of subjective judgment. Future studies would benefit from the development and integration of objective, quantitative biomarkers to refine this categorization.

In conclusion, this study demonstrates that cerebral perfusion and microstructural alterations in the basal ganglia are less pronounced in response to ischemia compared to the temporal lobe. These findings suggest that IVIM-derived parameters offer complementary and unique information regarding tissue microvascular status beyond what is captured by traditional hemodynamic perfusion imaging, potentially enhancing the comprehensive evaluation of ischemic pathophysiology.

## Supplementary Information

Below is the link to the electronic supplementary material.

Supplementary Material 1

## Data Availability

The datasets used and/or analyzed during the current study are available from the corresponding author on reasonable request.
