# Supplementary material for: Regional disparities in cerebral perfusion and brain tissue microstructure damage in adult patients with Moyamoya syndrome
Source: Sci Rep. 2025 Dec 1;16:995. doi: 10.1038/s41598-025-30486-4 (PMC12783861; doi:10.1038/s41598-025-30486-4)
Supplement: Supplementary file 1 — Supplementary Material 1 [file 41598_2025_30486_MOESM1_ESM.docx]

Table S1. Definition of Ischemia Grades in Moyamoya Syndrome

| **Grade** | **Name** | **Patient Level** | **Hemisphere Level** |
| --- | --- | --- | --- |
| 0 | **Normal** | Not applicable | A hemisphere in a patient with unilateral MMS where the major vessels are normal (no stenosis/occlusion). |
| **1** | **Asymptomatic** | Patient diagnosed with MMS during a routine examination, **presenting with no clinical symptoms**. | A hemisphere showing major vessel involvement (stenosis/occlusion) but no associated clinical symptoms or infarction lesions. |
| **2** | **Atypical** | Patient experiences non-specific symptoms such as **dizziness, headache, facial or limb discomfort**, but these **do not meet** the diagnostic criteria for TIA or cerebral infarction. | A hemisphere belonging to a patient with non-localizable, atypical clinical symptoms. |
| **3** | **Typical Ischemia** | Patient presents with either: 1) focal neurological deficits AND a recent infarct lesion on DWI; or 2) classic symptoms of TIA. Symptoms must be combined with involvement of major vessels in the culprit hemisphere.involvement of major vessels in the culprit hemisphere. | "A 'culprit hemisphere' causing the patient's typical ischemic symptoms (focal deficits or TIA), with confirmed involvement of its major vessels. |

**Table S2. ICC analysis of IVIM parameters between two readers.**

| ****Parameter**** | ****ICC Value**** | ****95% CI**** | ****Agreement Level**** |
| --- | --- | --- | --- |
| ****Temporal lobe**** | | | |
| ADC (×10^-4^ mm^2^/s) | 0.834 | 0.715 - 0.906 | Good |
| D (×10^-4^ mm^2^/s) | 0.881 | 0.792 - 0.933 | Good |
| D* (×10^-2^ mm^2^/s) | 0.906 | 0.835 - 0.948 | Excellent |
| f (×10^-1^) | 0.838 | 0.722 - 0.908 | Good |
| ****Basal Ganglia**** | | | |
| ADC (×10^-4^ mm^2^/s) | 0.803 | 0.666 - 0.887 | Good |
| D (×10^-4^ mm^2^/s) | 0.896 | 0.817 - 0.942 | Good |
| D* (×10^-2^ mm^2^/s) | 0.895 | 0.816 - 0.942 | Good |
| f (×10^-1^) | 0.846 | 0.735 - 0.913 | Good |

IVIM, intravoxel incoherent motion; f, perfusion fraction; D, diffusion; D*, pseudo-difusion; ADC, apparent difusion coefcient; ICC, Intraclass Correlation Coefficient; ICC was interpreted as follows: < 0.5 (poor), 0.5–0.75 (moderate), 0.75–0.9 (good), and > 0.9 (excellent).

Table S3. ICC analysis of CTP parameters between two readers.

| ****Parameter**** | ****ICC Value**** | ****95% Cl**** | ****Agreement Level**** |
| --- | --- | --- | --- |
| ****Temporal lobe**** | | | |
| **rCBV** | 0.840 | 0.725 - 0.909 | Good |
| **rCBF** | 0.882 | 0.793 - 0.934 | Good |
| **rMTT** | 0.851 | 0.742 - 0.916 | Good |
| **rTTP** | 0.805 | 0.669 - 0.888 | Good |
| ****Basal Ganglia**** | | | |
| **rCBV** | 0.858 | 0.755 - 0.920 | Good |
| **rCBF** | 0.871 | 0.776 - 0.927 | Good |
| **rMTT** | 0.833 | 0.713 - 0.905 | Good |
| **rTTP** | 0.902 | 0.827 - 0.945 | Excellent |

CTP, CT perfusion; rCBV, relative cerebral blood volume; rCBF, relative cerebral blood flow; rMTT, relative mean transit time; rTTP, relative time to peak; ICC, Intraclass Correlation Coefficient; ICC was interpreted as follows: < 0.5 (poor), 0.5–0.75 (moderate), 0.75–0.9 (good), and > 0.9 (excellent).

Table S4. Post hoc pairwise comparisons of parameters with significant overall differences across ischemia grades (Dunn's Test).

|  | **Temporal lobe** | | | | | | | | | | **Basal ganglia** | |
| --- | --- | --- | --- | --- | --- | --- | --- | --- | --- | --- | --- | --- |
| **Ischemia Grade** | **rMTT** | | **rTTP** | | **ADC** | | **D*** | | **f** | | **rTTP** | |
|  | Z value | *P* value | Z value | *P* value | Z value | *P* value | Z value | *P* value | Z value | *P* value | Z value | *P* value |
| **0 vs 1** | -4.771 | 0.417 | -3.110 | 0.002^#^ | 0.254 | 0.799 | -1.190 | 0.234 | 1.390 | 0.165 | -2.682 | 0.022^#^ |
| **0 vs 2** | -13.495 | 0.025^#^ | -2.142 | 0.032^#^ | 0.567 | 0.571 | -2.410 | 0.016^#^ | 2.415 | 0.016^#^ | -2.496 | 0.025^#^ |
| **0 vs 3** | -16.905 | 0.009^#^ | -3.580 | < 0.001^#^ | 3.172 | 0.002^#^ | 2.389 | 0.018^#^ | -2.471 | 0.013^#^ | -3.212 | 0.008^#^ |
| **1 vs 2** | -8.723 | 0.073 | 1.106 | 0.269 | 0.210 | 0.834 | 0.949 | 0.343 | -0.576 | 0.565 | 0.152 | 0.879 |
| **1 vs 3** | -12.133 | 0.025^#^ | -0.933 | 0.367 | 2.423 | 0.015^#^ | 1.156 | 0.248 | -0.834 | 0.404 | -0.927 | 0.425 |
| **2 vs 3** | -3.410 | 0.540 | -1.844 | 0.065 | 2.589 | 0.010^#^ | 0.365 | 0.716 | -0.371 | 0.710 | -1.034 | 0.452 |

Data are presented as Z value / *P* value. rMTT, relative mean transit time; rTTP, relative time to peak; ADC, apparent difusion coefcient; D*, pseudo-difusion; f, perfusion fraction; post hoc pairwise comparisons (Dunn's test with Bonferroni correction) were only performed for parameters that yielded a significant result (*p* < 0.05) in the overall Kruskal-Wallis test; ^#^ indicate statistically significant differences after Bonferroni correction at the 0.05 level.

Table S5. Post-hoc Power Analysis of CTP Parameters Stratified by Brain Region.

| **Parameter** | **P-value** | **Effect Size (ε²)** | **Power** | **Interpretation** |
| --- | --- | --- | --- | --- |
| **Temporal Lobe** | | | | |
| rCBV | 0.547 | 0.016 | 9.2% | **Likely True Negative.** |
| rCBF | 0.093 | 0.085 | 33.3% | **High risk of Type II error (False Negative).** |
| rMTT | **0.018*** | 0.177 | 68.7% | Adequate power for a medium effect. |
| rTTP | **0.002*** | 0.288 | **93.6%** | High power confirms a true positive result. |
| ****Basal Ganglia**** | | | | |
| rCBV | 0.669 | 0.007 | 6.7% | **Likely True Negative.** |
| rCBF | 0.650 | 0.018 | 9.7% | **Likely True Negative.** |
| rMTT | 0.217 | 0.061 | 24.1% | **Likely True Negative.** |
| rTTP | **0.011*** | 0.205 | **77.4%** | Good power supports this positive result. |

CBV, cerebral blood volume; CBF, cerebral blood flow; MTT, mean transit time; TTP, time to peak; ADC, apparent diffusion coefficient; D, diffusion; D*, pseudo-diffusion; f, perfusion fraction; Effect size (Epsilon-squared, ε²) quantifies the magnitude of the difference between groups, complementing the p-value by indicating its practical importance: ε² ≥ 0.26 (Large), 0.08-0.26 (Medium), 0.01-0.08 (Small), and <0.01 (Negligible).

Table S6. Post-hoc Power Analysis of IVIM Parameters Stratified by Brain Region.

| **Parameter** | **P-value** | **Effect Size (ε²)** | **Power** | **Interpretation** |
| --- | --- | --- | --- | --- |
| **Temporal Lobe** | | | | |
| ADC | **0.011*** | 0.206 | **77.6%** | Good power supports this positive result. |
| D | 0.593 | 0.023 | 11.1% | **Likely True Negative.** |
| D* | **0.041*** | 0.132 | 52.3% | Modest power; a true positive but requires cautious interpretation. |
| f | **0.043*** | 0.128 | 50.7% | Modest power; a true positive but requires cautious interpretation. |
| ****Basal Ganglia**** | | | | |
| ADC | 0.464 | 0.039 | 16.2% | **Likely True Negative.** |
| D | 0.761 | 0.004 | 5.9% | **Likely True Negative.** |
| D* | 0.162 | 0.078 | 30.7% | **High risk of Type II error (False Negative).** |
| f | 0.256 | 0.064 | 25.1% | **High risk of Type II error (False Negative).** |

CBV, cerebral blood volume; CBF, cerebral blood flow; MTT, mean transit time; TTP, time to peak; ADC, apparent diffusion coefficient; D, diffusion; D*, pseudo-diffusion; f, perfusion fraction; Effect size (Epsilon-squared, ε²) quantifies the magnitude of the difference between groups, complementing the p-value by indicating its practical importance: ε² ≥ 0.26 (Large), 0.08-0.26 (Medium), 0.01-0.08 (Small), and <0.01 (Negligible).
